# Supplementary material for: Neuromotor changes in participants with a concussion history can be detected with a custom smartphone app
Source: PLoS One. 2022 Dec 15;17(12):e0278994. doi: 10.1371/journal.pone.0278994 (PMC9754195; doi:10.1371/journal.pone.0278994)
Supplement: Attachment — Submitted filename: Response to Reviewers.docx [file pone.0278994.s003.docx]

The authors would like to thank the reviewers for their thoughtful comments, as well as thanking the editor for the opportunity to resubmit our paper for publication consideration in PLOS ONE. We have attended to all of the reviewers’ comments/suggestions in the manuscript and/or in this document and have provided a point-by-point response to all comments/suggestions below. The reviewers’ comments are in **bold**, our responses are in normal typeface, and the text that has been added to the manuscript is in red.

**Journal Requirements comments**

1) **Line 260: Please correct ‘remained’ to ‘remaining’.**

Thank you for catching this typo. We have updated the text as requested.

2) **Table 3: Per our statistical reporting guidelines (https://journals.plos.org/plosone/s/submission-guidelines.#loc-statistical-reporting), please report exact p-values for all values greater than or equal to 0.001. P-values less than 0.001 may be expressed as p < 0.001, or as exponentials in studies of genetic associations.**

We have updated this table to align with the reporting guidelines.

3) **Line 424: Please capitalise ‘We’.**

Thank you for catching this typo. We have updated the text as requested.

**Reviewer #1 Comments**

1) **The revision is good. It should be published. There are some extra characters to meet the minimum character count that is required.**

Thank you for your comment and your feedback throughout the peer-review process.

**Reviewer #3 Comments**

1) **Most importantly, this analysis shows a statistical association, not discriminative ability – to do that would require a classification workflow (e.g., held out test set, classification performance assessment). I would encourage clarification of the language throughout the manuscript on this point.**

Thank you for the suggestion and we agree with your comment. Accordingly, we revised the language to align with the analysis in the following manner:

In the Introduction and Discussion sections:

“The purpose of the current study was to examine the extent to which the neuromotor behavior data obtained from our smartphone app could associate with a history of concussion.”

In the Result section, we confirmed that the findings are expressed as statistical association. Lastly, in the Conclusion section, we have modified the text accordingly:

“Collectively, this study shows that neuromotor performance on an objective, cost-effective, and portable dynamic balance assessment is associated with a history of concussion.”

2) **Although the authors are to be commended for sharing the raw data from the study, it would help the reader to see descriptive analyses of the study variables – for example, a tabulation of the central tendency and distribution _and_ a scatter plot of the SD of maximum velocity (and the other ~6 potential input variables to a discriminative tool), across concussion vs. no concussion groups.**

We appreciate this suggestion. Accordingly, have added the descriptive table and plots in the raw data file as requested. Because of the non-interaction, the head-shake condition is displayed. This information is also pasted below for ease of review.

**Specific comments**

3) **Both civilians and service members were young (18-50), healthy, and fit (height/weight). These results may not generalize to different populations.**

Thank you for bringing up this important point. To acknowledge it in the manuscript, we have added the following limitation to the last paragraph of the Discussion section.

“Lastly, the participants in this study could be generally characterized and young, fit, and physically active. Thus, these results may not generalize to populations outside of this characterization.”

4) **For subgroups among whom 3 trials were performed, only 2 were analyzed. How were the 2/3 selected?**

Thank you for bringing up this important point. Only the first 2 trials were used (even if 3 trials were available) in our analyses to ensure an equitable comparison among the groups. We have clarified this as follows in the text.

“To compare common data that were collected at all sites, the mean of all dependent variables for the first 2 trials (instead of all 3 trials) were calculated for the EC and HS conditions, which were then submitted for statistical analysis.”

5) **Was participant history of musculoskeletal injury collected? Such injuries could affect both range of motion and balance.**

Unfortunately, these data were not collected at all sites, so neither a descriptive nor a general comment on past musculoskeletal injury could be included in the analyses or discussion.

6) **Table 1 and Results (first paragraph) – it is generally useful to have key comparisons be shown between table columns, with p-values shown in the table rather than only in text.**

Thank you for this suggestion. We have added a new column that includes p-values for our primary comparison groups (healthy vs. concussed) relative to height and weight.

| **Demographics by Group (Civilians and Service Members Collapsed)** | | | |  |
| --- | --- | --- | --- | --- |
| **Category** | Healthy  (n=154) | Concussion History (n=62) | p-value |  |
| **Height (cm)** | 171.99 ± 9.70 | 171.43 ± 12.59 | 0.749 |  |
| **Weight (kg)** | 70.93 ± 13.82 | 75.26 ± 16.19 | 0.072 |  |
| **Time from concussion to testing (weeks)** | N/A | 10.13 ± 8.67 | N/A |  |
| **Healthy and Concussion History within Each Population** | | | | |
| **Population** | Civilians | | Service Members | |
| **Category** | Healthy (n=100) | Concussion History (n=44) | Healthy  (n = 54) | Concussion History (n=18) |
| **Height (cm)** | 171.99 ± 9.70 | 170.08   ± 13.87 | N/A | 174.72 ± 8.13 |
| **Weight (kg)** | 70.93 ± 13.82 | 73.75 ± 17.90 | N/A | 78.94 ± 13.46 |
| **Time from concussion to testing (weeks)** | N/A | 6.52 ± 6.32 | N/A | 18.16 ± 8.15 |

7) **The time from concussion to testing was quite different between the two populations (6+ weeks civilians, ~18 weeks military) in a way that could affect results. Statistical associations were not found, but a more standardized study would be necessary before any final conclusions were drawn.**

Thank you for your comments. We originally added the variable in the model, but the association was too low, and therefore, we decided to omit the variable. However, we agree that this information is important. Thus, we have added an additional graphic (Figure 4) and text to the manuscript to address this point. The following red text has been added to the last paragraph in the Discussion section.

“There are several limitations of this study to acknowledge. First, although the inclusion criteria required the concussive event to have occurred no longer than six months prior to our testing, the number of weeks since the event varied substantially in our population. Concussion symptoms can dissipate for many people 7-10 days after the event, so future work should explore the factor of time-since-injury as part of their study design. Prior to the main analysis, we had run a correlation analysis within the concussion history group between the dependent variable and time from concussion (n = 54; time from concussion was missing for n = 8). The results of the Spearman correlation did not show a significant association (rho = - 0.013, p = 0.888) (Figure 4). Thus, we did not include this variable in our model. However, it is possible that this characteristic is unique to our cohort. More research is warranted to understand movement variability and the time from concussion.”
